# Social Welfare Control in Mobile Crowdsensing Using Zero-Determinant Strategy

**DOI:** 10.3390/s17051012

**Published:** 2017-05-03

**Authors:** Qin Hu, Shengling Wang, Rongfang Bie, Xiuzhen Cheng

**Affiliations:** 1 College of Information Science and Technology, Beijing Normal University, Beijing 100875, China; huqin@mail.bnu.edu.cn (Q.H.); rfbie@bnu.edu.cn (R.B.); 2 Department of Computer Science, George Washington University, Washington, DC 20052, USA; cheng@gwu.edu

**Keywords:** crowdsensing, game theory, zero-determinant strategy, social welfare

## Abstract

As a promising paradigm, mobile crowdsensing exerts the potential of widespread sensors embedded in mobile devices. The greedy nature of workers brings the problem of low-quality sensing data, which poses threats to the overall performance of a crowdsensing system. Existing works often tackle this problem with additional function components. In this paper, we systematically formulate the problem into a crowdsensing interaction process between a requestor and a worker, which can be modeled by two types of iterated games with different strategy spaces. Considering that the low-quality data submitted by the workers can reduce the requestor’s payoff and further decrease the global income, we turn to controlling the social welfare in the games. To that aim, we take advantage of zero-determinant strategy, based on which we propose two social welfare control mechanisms under both game models. Specifically, we consider the requestor as the controller of the games and, with proper parameter settings for the to-be-adopted zero-determinant strategy, social welfare can be optimized to the desired level no matter what strategy the worker adopts. Simulation results demonstrate that the requestor can achieve the maximized social welfare and keep it stable by using our proposed mechanisms.

## 1. Introduction

With the rapid development of micro-electro-mechanical systems and digital electronics, more and more functional sensors (e.g., accelerometers, cameras and compasses) are equipped in various kinds of mobile devices (e.g., smartphones and wearable devices). On the other hand, the development of wireless communication technologies (e.g., bluetooth and Wi-Fi) has stepped into a new era. These advances contribute substantially to the emergence of mobile crowdsensing [1,2,3]. Unlike traditional sensor networks with numerous static sensors [4,5], mobile crowdsensing takes advantage of the massive mobile-device owners (i.e., workers) and their mobility to obtain comprehensive and real-time sensing outcomes for requestors. However, since workers with different backgrounds and skills often undertake tasks without strict rules, they may provide low-quality data (e.g., fabricated or incomplete data) to get more profit. This situation is harmful to requestors in a short term and is disastrous to the whole crowdsensing system in a long term. Therefore, it is imperative to tackle the problem of low-quality data generated by the workers in mobile crowdsensing.

Recently, plenty of research focusing on data quality in mobile crowdsensing has been presented. *Auction*, a powerful tool in economics, has been widely adopted to ensure data quality. Accordingly, auctions are employed in the design of incentive mechanisms for mobile crowdsensing [6,7,8]. However, the negotiation process in an auction usually costs excessive time, which is redundant and cumbersome for lightweight mobile crowdsensing applications. History behaviorial information of the participants also has been utilized. Crowdsensing quality was improved by filtering out low-quality data providers in the reputation-based-schemes [9] and machine-learning-based-schemes [10]. Nevertheless, such an approach may fail due to the lack of sufficient training or history data.

To conquer the existing issues in mobile crowdsensing quality control, we devise a more cost-efficient and systematic approach in this paper. Our approach embeds a data quality improvement process into crowdsensing interaction. We first formulate the interaction between the requestor and any worker in mobile crowdsensing as an iterated game. In this game, the payment amount is regarded as the strategy of the requestor, and the submitted data quality is the action of the worker. Note that the worker’s bad actions can directly lower the requestor’s profit, and can bring damage to the total profit of the crowdsensing system. As the requestor is the victim of the low-quality data problem as well as the recruiter of the crowdsensing task, we consider that the requestor has the responsibility and capability to deal with the bad actions. Therefore, in order to warrant mobile crowdsing quality effectively and efficiently, we propose social welfare controlling schemes for the requestor to guarantee the whole interest of the crowdsensing system regardless of the workers’ actions.

More specifically, we analyze the problem under two situations with different types of strategies (i.e., *discrete strategy* and *continuous strategy*) a requestor and a worker may take. As a result, we propose a zero-determinant strategy that offers the possibility for the requestor to unilaterally control the social welfare in the iterated game. By solving a constrained optimization problem, we design social welfare control mechanisms for the two models. The requestor only needs to setup appropriate parameters for her (In this paper, we denote the requestor as “she” and the worker as “he” for better distinction) strategy, so as to obtain the desired social welfare instead of sparing any effort to deal with the workers’ actions. In other words, with the proposed schemes for the requestor, a worker’s strategy has no impact on the social welfare.

Concentrating on solving the above problem in mobile crowdsensing, we summarize the contributions of this paper as follows:

- We make use of two types of iterated games to formulate the interactions between the requestor and the worker in mobile crowdsensing when they adopt discrete strategies and continuous strategies.
- Based on the original zero-determinant strategy derived under the discrete model, we extend it with sufficient theoretic derivation to make it applicable for the continuous model. Note that this extension not only provides theoretical foundation for our proposed mechanism but also expands the application range of zero-determinant strategy.
- We propose two social welfare control mechanisms for the requestor under two different situations, which helps the requestor establish an overall control over the quality of the whole crowdsensing system so as to solve the low-quality data problem from the perspective of the ultimate goal.

The rest of the paper is organized as follows. Section 2 formulates the games between the requestor and any worker in mobile crowdsensing under the discrete-strategy and continuous-strategy situations. The mechanisms achieving social welfare control under two different models are proposed in Section 3. We simulate our proposed schemes in Section 4 to verify their effectiveness. Most related works are investigated in Section 5 and the whole paper is concluded in Section 6.

## 2. Game Formulation

In this paper, we consider the following crowdsensing scenario. As the initiator of mobile crowdsensing tasks, the requestor publishes jobs with the corresponding payments. The workers claim the jobs that they can accomplish in order to get payments. At each round, the requestor can distribute different payments to a certain job while the workers can choose to provide different levels of task quality to the claimed jobs. With the temptation of getting more profit, the workers may maliciously provide very low job quality. Because of the limitation on the resources of a requestor (e.g., computing power and detection time), we assume that the requestor has difficulty to discover the bad behavior of a worker in a timely manner. However, the requestor is able to find out a worker’s malicious behavior of providing low job quality after a certain number of rounds and then penalize the worker in future rounds. The above working scenario between the requestor and any one of the workers can be viewed as a game; and it turns out to be an iterated game if the same worker is recruited in multiple rounds.

For the iterated game mentioned above, the strategy of the requestor is considered to be the amount of payment she determines to offer to the worker for a specific job, while the strategy of the worker is the quality of the job he decides to provide in the following job completing process. Since the strategies of both players could be either simple or complicated, we consider them as either discrete or continuous. A discrete strategy means that both players adopt either an extremely amicable or a vicious action, while a continuous one refers to any action as long as it is in the corresponding continuous strategy space. The distinction on strategy types may result in totally different problem formulations and solutions in the iterated game; thus we model the above problem by considering a discrete model and a continuous model.

In the discrete model, the requestor can choose the strategy of providing the highest or the lowest payment for a certain job, and the worker determines his strategy on providing the highest or the lowest quality for accomplishing the claimed job. Here, the requestor’ strategy is defined as x∈{c,d}, where *c* is the abbreviation of cooperation denoting the highest payment and *d* means defection referring to the lowest payment; and the worker’s strategy is y∈{c,d}, where *c* refers to the highest job quality and *d* is the lowest. At each round, the strategy that any player adopts is private; thus there could be four outcomes of the game between the requestor and the worker, i.e., xy=(cc,cd,dc,dd).

**Figure 1:**
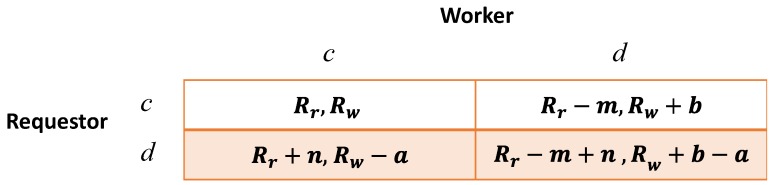
Payoffs of the requestor and the worker at each round.

We define $R_{r}$ and $R_{w}$ as the payoff of the requestor and that of the worker when they mutually cooperate. Let *b* be the increase on payoff when the worker defects but the requestor cooperates, in which case the requester gets a decrease *m* on payoff; let *n* be the increase on payoff when the requestor defects but the worker cooperates, and thus the worker’s payoff will have a decrease *a*. Here, we consider n<m,b<a because the lowest payment of the requestor and the lowest job quality of the worker could result in less payoff for both of them than that of the case when both cooperate. We denote the payoff vector of the requestor as $S_{r}=\left(S_{r}^{1},S_{r}^{2},S_{r}^{3},S_{r}^{4}\right)=\left(R_{r},R_{r}-m,R_{r}+n,R_{r}-m+n\right)$ and that of the worker as $S_{w}=\left(S_{w}^{1},S_{w}^{2},S_{w}^{3},S_{w}^{4}\right)=\left(R_{w},R_{w}+b,R_{w}-a,R+b-a\right)$ under the state xy=(cc,cd,dc,dd), which are demonstrated in Figure 1. For the requestor, it is obvious that no matter what the worker’s strategy is, her best strategy is x=d; and, similarly, the worker can also derive that his best strategy is y=d. Thus, this game has an equilibrium of xy=dd, where the social welfare $S_{r}^{4}+S_{w}^{4}$ is certainly less than that of any other state.

In the continuous model, the requestor can choose any payment from her strategy space, while the worker can determine any job quality from his strategy domain. We denote the requestor’s strategy as $x\in [l_{r},h_{r}]$, where $l_{r}$ and $h_{r}$ are respectively the lowest and the highest payment that the requestor can offer; and the worker’s strategy is denoted as $y\in [l_{w},h_{w}]$, where $l_{w}$ and $h_{w}$ are respectively the lowest and the highest job quality the worker can provide.

We define the requestor’s utility as

(1) $$w_{r}\left(x,y\right)=A_{r}\phi \left(y\right)-B_{r}x,$$

where the first term is the profit that the accomplished job brings to the requestor and the second term refers to the payment she should assign to the worker; the scaling parameters $A_{r}>0$ and $B_{r}>0$; and ϕ(y) monotonically increases with the worker’s strategy *y*.

With a similar composition, the worker’s utility can be expressed as

(2) $$w_{w}\left(x,y\right)=A_{w}x-B_{w}\psi \left(y\right),$$

where the first term is the payment the worker can acquire by completing the sensing job, while the second term reflects the cost in the job completing process. The scaling parameters $A_{w}>0$ and $B_{w}>0$; and ψ(y) also monotonically increases with *y*.

To briefly examine the equilibrium of this continuous game, we make the following calculations. For the requestor, we have $\frac{\partial w_{r}\left(x,y\right)}{\partial x}=-B_{r}<0$, which means that her utility decreases with *x*; thus the requestor’s best strategy is $x^{*}=l_{r}$ no matter what the worker’s strategy *y* is. On the other hand, since ψ(y) increases as *y* increases, we have $\psi^{\prime }\left(y\right)>0$. Combining with $B_{w}>0$, we have $\frac{\partial w_{w}\left(x,y\right)}{\partial y}=-B_{w}\psi^{\prime }\left(y\right)<0$, which means that the worker can get his best strategy $y^{*}=l_{w}$ regardless of the requestor’s strategy *x*. Therefore, the stable equilibrium of the continuous-model game is $\left(x^{*},y^{*}\right)=\left(l_{r},l_{w}\right)$, which is clearly an unexpected outcome for the requestor.

## 3. Game Analysis and Mechanism Design

Based on the analysis in Section 2, one can see that the stable equilibrium states in both the discrete and the continuous models are unfavorable to the requestor. Moreover, these models are certainly inefficient for both the requestor and the worker in a short term, and it is potentially harmful to the stability and sustainability of the crowdsensing system. Thus, it is necessary for the requestor to address the issue as she is the sensing task employer who is assumed to have the responsibility and capability for solving such a problem. Therefore, in this section, we first provide a further analysis on the iterated games under these two different models and then propose mechanisms to help the requestor control the social welfare in mobile crowdsensing without considering the worker’s specific strategy.

### 3.1. Zero-Determinant Strategy Based Scheme in the Discrete Model

In this paper, we assume that all the players only have the memory of the state in the last round. As mentioned in [11], a short-memory player rather than a long-memory player determines the rules of the game. In our game, both players have mixed strategies at each round denoting the cooperation probabilities under the four possible states in the last round. Accordingly, we define the mixed strategy of the requestor as $p^{t}=\left(p_{1}^{t},p_{2}^{t},p_{3}^{t},p_{4}^{t}\right)$ and that of the worker as $q^{t}=\left(q_{1}^{t},q_{2}^{t},q_{3}^{t},q_{4}^{t}\right)$. Here, $p_{1}^{t},\;p_{2}^{t},\;p_{3}^{t},\;p_{4}^{t}$ and $q_{1}^{t}$, $q_{2}^{t}$, $q_{3}^{t}$, $q_{4}^{t}$ are the probabilities of choosing *c* in round *t* when the outcome of round t−1 is xy=cc,cd,dc,dd. Additionally, we denote the possibilities of the four potential states at round *t* as $v^{t}=\left[v_{1}^{t},v_{2}^{t},v_{3}^{t},v_{4}^{t}\right]$, where $\sum_{i=1}^{4}v_{i}=1$; thus the corresponding payoffs of the requestor and the worker are $E_{r}^{t}=v^{t}S_{r}$ and $E_{w}^{t}=v^{t}S_{w}$, respectively.

With the definitions of $p^{t}$ and $q^{t}$ mentioned above, one can get the Markov state transition matrix as follows,

(3) $$\begin{array}{c} M=\left[\begin{array}{cccc} p_{1}^{t}q_{1}^{t} & p_{1}^{t}\left(1-q_{1}^{t}\right) & \left(1-p_{1}^{t}\right)q_{1}^{t} & \left(1-p_{1}^{t}\right)\left(1-q_{1}^{t}\right)\\ p_{2}^{t}q_{2}^{t} & p_{2}^{t}\left(1-q_{2}^{t}\right) & \left(1-p_{2}^{t}\right)q_{2}^{t} & \left(1-p_{2}^{t}\right)\left(1-q_{2}^{t}\right)\\ p_{3}^{t}q_{3}^{t} & p_{3}^{t}\left(1-q_{3}^{t}\right) & \left(1-p_{3}^{t}\right)q_{3}^{t} & \left(1-p_{3}^{t}\right)\left(1-q_{3}^{t}\right)\\ p_{4}^{t}q_{4}^{t} & p_{4}^{t}\left(1-q_{4}^{t}\right) & \left(1-p_{4}^{t}\right)q_{4}^{t} & \left(1-p_{4}^{t}\right)\left(1-q_{4}^{t}\right) \end{array}\right], \end{array}$$

where each element denotes the probability of the state transferring from round t−1 to *t*. Here, each row of (3) corresponds to the state at round t−1 following the order xy=cc,cd,dc,dd and each column is corresponding to the state at round *t* following the same order. For example, $M_{23}$ denotes the transition probability from state xy=cd at round t−1 to the state xy=dc at round *t*.

We assume that the stable vector of the above transition matrix M is denoted by $v_{s}^{T}$; thus we have

(4) $$v_{s}^{T}M=v_{s}^{T}.$$

Inspired by the calculation in [11], we denote by I the unitary matrix, and let $M^{\prime }=M-I$; thus we have $v_{s}M^{\prime }=0.$ Then, we can obtain $Adj\left(M^{\prime }\right)M^{\prime }=det\left(M^{\prime }I\right)=0$ according to the Cramer’s rule, in which $Adj(M^{\prime })$ denotes the adjugate matrix of $M^{\prime }$. Comparing the above two equations, one can see that v is proportional to every row of $Adj(M^{\prime })$. Therefore, the dot product of the stable vector $v_{s}$ and any vector $f=(f_{1},f_{2},f_{3},f_{4})$ can be calculated as follows:

(5) $$\begin{array}{cc} v_{s}^{T}\cdot f & =D(p^{t},q^{t},f),\\ & =det\left[\begin{array}{cccc} p_{1}^{t}q_{1}^{t}-1 & p_{1}^{t}-1 & q_{1}^{t}-1 & f_{1}\\ p_{2}^{t}q_{3}^{t} & p_{2}^{t}-1 & q_{2}^{t} & f_{2}\\ p_{3}^{t}q_{3}^{t} & p_{3}^{t} & q_{3}^{t}-1 & f_{3}\\ p_{4}^{t}q_{4}^{t} & p_{4}^{t} & q_{4}^{t} & f_{4} \end{array}\right]. \end{array}$$

Notably, the second column of (5) can be determined by the requestor alone, which is denoted as ${\tilde{p}}^{t}={\left(p_{1}^{t}-1,p_{2}^{t}-1,p_{3}^{t},p_{4}^{t}\right)}^{T}$. Hence, when $f=\alpha S_{r}+\beta S_{w}+\gamma 1$, we have

(6) $$v_{s}^{T}\cdot f=v_{s}^{T}\cdot \left(\alpha S_{r}+\beta S_{w}+\gamma 1\right)=\alpha E_{r}+\beta E_{w}+\gamma ,$$

where $E_{r}$ and $E_{w}$ are, respectively, the expected payoffs of the requestor and the worker in the final stable state; α,β,γ are scalars. Based on (5), we also have

(7) $$\alpha E_{r}+\beta E_{w}+\gamma =D\left(p^{t},q^{t},\alpha S_{r}+\beta S_{w}+\gamma 1\right).$$

Therefore, if the requestor selects strategy $p^{t}$ satisfying ${\tilde{p}}^{t}=\chi \left(\alpha S_{r}+\beta S_{w}+\gamma 1\right)\left(\chi \neq 0\right)$, the corresponding matrix’s second column is proportional to the fourth column, which implies that the above equation is equal to zero, i.e., $\alpha E_{r}+\beta E_{w}+\gamma =0$. Therefore, the strategy adopted by the requestor is known as a zero-determinant strategy. Then the weighted *social welfare* of this game can be defined as

(8) $$E_{all}=\alpha E_{r}+\beta E_{w}=-\gamma .$$

The above analysis indicates that when the requestor adopts a zero-determinant strategy, she can have a unilateral control over the social welfare, which can be fixed to a desired value no matter what strategy the worker adopts. This provides the requestor a powerful tool to maintain the stability and sustainability of the crowdsensing system.

In order to set the optimum and stable social welfare regardless of the worker’s strategy, the requestor needs to solve the following constrained optimization problem:

(9) $$\begin{array}{cc} \max & \;\;E_{all}=\alpha E_{r}\left(p^{t},q^{t}\right)+\beta E_{w}\left(p^{t},q^{t}\right),\forall q^{t},\\ s.t. & \left\{\begin{array}{c} 0\leq p^{t}\leq 1,\\ \alpha E_{r}+\beta E_{w}+\gamma =0. \end{array}\right. \end{array}$$

As mentioned in (8), it is equivalent to solve the following problem:

(10) $$\begin{array}{cc} \min & \;\;\gamma ,\\ s.t. & \left\{\begin{array}{c} 0\leq p^{t}\leq 1,\\ {\tilde{p}}^{t}=\chi \left(\alpha S_{r}+\beta S_{w}+\gamma 1\right),\\ \chi \neq 0. \end{array}\right. \end{array}$$

For the case of χ>0, when considering the constraint $p^{t}\geq 0$, one can get

(11) $$\begin{array}{cc} \gamma_{min} & =\max\left(\Gamma_{i}\right),\forall i\in \left\{1,2,3,4\right\},\\ \Gamma_{i} & =\left\{\begin{array}{cc} -\alpha S_{r}^{i}-\beta S_{w}^{i}-\frac{1}{\chi }, & i=1,2,\\ -\alpha S_{r}^{i}-\beta S_{w}^{i}, & i=3,4. \end{array}\right. \end{array}$$

while when considering the constraint condition $p^{t}\leq 1$, we have

(12) $$\begin{array}{cc} \gamma_{max} & =\min\left(\Gamma_{j}\right),\forall j\in \left\{5,6,7,8\right\},\\ \Gamma_{j} & =\Gamma_{i+4}=\left\{\begin{array}{cc} -\alpha S_{r}^{i}-\beta S_{w}^{i}, & i=1,2,\\ -\alpha S_{r}^{i}-\beta S_{w}^{i}+\frac{1}{\chi }, & i=3,4. \end{array}\right. \end{array}$$

Note that γ has a feasible solution only when it meets $\gamma_{min}<\gamma_{max}$, i.e., $\max\left(\Gamma_{i}\right)<\min\left(\Gamma_{j}\right),\forall i\in \left\{1,2,3,4\right\},\forall j\in \left\{5,6,7,8\right\}$. Considering that χ could be any positive value, we can obtain the minimum value of γ as follows,

(13) $$\gamma_{min}=\max\left(-\alpha S_{r}^{3}-\beta S_{w}^{3},-\alpha S_{r}^{4}-\beta S_{w}^{4}\right).$$

For the case of χ<0, when considering that $p^{t}\geq 0$, we have $\gamma_{min}=\max\left(\Gamma_{j}\right),\forall j\in \left\{5,6,7,8\right\}$; while when considering that $p^{t}\leq 1$, we have $\gamma_{max}=\min\left(\Gamma_{i}\right),\forall i\in \left\{1,2,3,4\right\}$. Then, γ is feasible when $\gamma_{min}<\gamma_{max}$, i.e., $\max\left(\Gamma_{j}\right)<\min\left(\Gamma_{i}\right),\forall i\in \left\{1,2,3,4\right\},\forall j\in \left\{5,6,7,8\right\}$. Finally, we can get the following result:

(14) $$\gamma_{min}=\max\left(-\alpha S_{r}^{1}-\beta S_{w}^{1},-\alpha S_{r}^{2}-\beta S_{w}^{2}\right).$$

According to (13) and (14), when the requestor adopts the zero-determinant strategy $p^{t}$ meeting ${\tilde{p}}^{t}=\chi \left(\alpha S_{r}+\beta S_{w}+\gamma 1\right)$, each element of $p^{t}$ can be calculated as follows:

(15) $$\begin{array}{c} p_{i}^{t}=\left\{\begin{array}{cc} \chi (\alpha S_{r}^{i}+\beta S_{w}^{i}+\gamma_{min})+1, & i=1,2,\\ \chi (\alpha S_{r}^{i}+\beta S_{w}^{i}+\gamma_{min}), & i=3,4. \end{array}\right. \end{array}$$

### 3.2. Zero-Determinant Strategy Based Scheme in the Continuous Model

In order to solve the problem under the continuous model, we also assume that both the requestor and the worker make their choices on strategies according to the outcome of the last round. Similarly, we define the mixed strategy of the requestor $p^{t}\left(x_{-1},y_{-1},x\right)$ as the probability she chooses the payment *x* at round *t* when the state at round t−1 is $x_{-1}y_{-1}$, where $x_{-1},x\in \left[l_{r},h_{r}\right]$ and $y_{-1}\in \left[l_{w},h_{w}\right]$. Since the strategy *x* can be any value in the continuous domain, we have

(16) $$\int_{l_{r}}^{h_{r}}p^{t}\left(x_{-1},y_{-1},x\right)dx=1.$$

In addition, the mixed strategy of the worker $q^{t}\left(x_{-1},y_{-1},y\right)$ also refers to the conditional probability he provides job quality *y* when the state at the last round is $x_{-1}y_{-1}$, where $x\in [l_{r},h_{r}]$ and $y_{-1},y\in \left[l_{w},h_{w}\right]$. There also exists the following relationship:

(17) $$\int_{l_{w}}^{h_{w}}q^{t}\left(x_{-1},y_{-1},y\right)dy=1.$$

Next, we suppose that the joint probability that the requestor adopts strategy *x* and the worker adopts *y* at each round is v(x,y). Considering the utility functions $w_{r}\left(x,y\right),w_{w}\left(x,y\right)$, one can get the expected utility of the requestor and that of the worker at round *t* as follows:

(18) $$E_{r}^{t}=\int_{l_{w}}^{h_{w}}\int_{l_{r}}^{h_{r}}v\left(x,y\right)w_{r}\left(x,y\right)dxdy,$$

(19) $$E_{w}^{t}=\int_{l_{w}}^{h_{w}}\int_{l_{r}}^{h_{r}}v\left(x,y\right)w_{w}\left(x,y\right)dxdy.$$

Similar to the state transition matrix M in the discrete model, there is a transition function, denoted as $M(x_{-1},y_{-1},x,y)$, indicating the state transition probability from round t−1 to round *t*, which can be expressed as:

(20) $$M\left(x_{-1},y_{-1},x,y\right)=p^{t}\left(x_{-1},y_{-1},x\right)q^{t}\left(x_{-1},y_{-1},y\right).$$

Then, the state probabilities at two sequential rounds have the following relationship:

(21) $$v\left(x_{-1},y_{-1}\right)\cdot M\left(x_{-1},y_{-1},x,y\right)=v\left(x,y\right).$$

With a similar analysis as the one for the zero-determinant strategy derived in the discrete model, we can figure out the zero-determinant strategy in the continuous model, which is summarized as follows.

**Lemma** **1.** *When the requestor’s strategy $p^{t}\left(x_{-1},y_{-1},x\right)$ satisfies ${\tilde{p}}^{t}\left(x_{-1},y_{-1},h_{r}\right)=\chi \left(\alpha w_{r}\left(x,y\right)+\beta w_{w}\left(x,y\right)+\gamma \right)\left(\chi \neq 0\right)$, the requestor’s expected utility $E_{r}$ in a stable state and that of the worker $E_{w}$ meet the following relationship:*

(22) $$\alpha E_{r}+\beta E_{w}+\gamma =0,$$

*where ${\tilde{p}}^{t}\left(x_{-1},y_{-1},h_{r}\right)$ is defined as*

(23) $$\begin{array}{c} {\tilde{p}}^{t}\left(x_{-1},y_{-1},h_{r}\right)=\left\{\begin{array}{cc} p^{t}\left(x_{-1},y_{-1},h_{r}\right),\; & x<h_{r},\\ p^{t}\left(x_{-1},y_{-1},h_{r}\right)-1,\; & x=h_{r}. \end{array}\right. \end{array}$$

**Proof.**  We first divide the continuous strategy space into η parts. Then the strategies of the requestor and the worker turn to be $x\in \{l_{r},l_{r}+\delta ,l_{r}+2\delta ,\cdots ,l_{r}+\eta \delta \}$, and $y\in \{l_{w},l_{w}+\delta ,l_{w}+2\delta ,\cdots ,l_{w}+\eta \delta \}$, respectively, where δ is sufficiently small while η is sufficiently large, satisfying $l_{r}+\eta \delta =h_{r}$ and $l_{w}+\eta \delta =h_{w}$. It is clear that when δ→0, the strategy space is approximately continuous. Accordingly, the payoffs of the requestor and the worker are $W_{r}=\left\{w_{r}\left(l_{r},l_{w}\right),\cdots ,w_{r}\left(l_{r},l_{w}+\eta \delta \right),\cdots ,w_{r}\left(l_{r}+\eta \delta ,l_{w}\right),\cdots ,w_{r}\left(l_{r}+\eta \delta ,l_{w}+\eta \delta \right)\right\}=\left\{w_{r00},\cdots ,w_{r0\eta },\cdots ,w_{r\eta 0},\cdots ,w_{r\eta \eta }\right\}$ and $W_{w}=\left\{w_{w}\left(l_{r},l_{w}\right),\cdots ,w_{w}\left(l_{r},l_{w}+\eta \delta \right),\cdots ,w_{w}\left(l_{r}+\eta \delta ,l_{w}\right),\cdots ,w_{w}\left(l_{r}+\eta \delta ,l_{w}+\eta \delta \right)\right\}=\left\{w_{w00},\cdots ,w_{w0\eta },\cdots ,w_{w\eta 0},\cdots ,w_{w\eta \eta }\right\}$, respectively.

Then, we define the requestor’s mixed strategy at round *t* as $p_{ij-k}^{t},\forall i,j,k\in \left\{0,1,\cdots ,\eta \right\}$, which means that the probability of the requestor choosing $x=l_{r}+k\delta$ at round *t* when the last state is $x_{-1}=l_{r}+i\delta ,y_{-1}=l_{w}+j\delta$; similarly, the worker’s mixed strategy at round *t* is $q_{ij-k}^{t}$, where i,j,k∈{0,1,⋯,η}.

According to the above division on the strategy space and utility space, we have a Markov state transition matrix as follows:

(24) $$M_{d}=\left[M_{00},\cdots ,M_{0\eta },M_{11},\cdots ,M_{1\eta },\cdots ,M_{\eta 0},\cdots ,M_{\eta \eta }\right],$$

where each element $M_{ij},\forall i,j\in \left\{0,1,\cdots ,\eta \right\}$ is a vector containing the transition probability from all the possible combinations of the last state $x_{-1}y_{-1}$ to the current state $x=l_{r}+i\delta$ and $y=l_{w}+j\delta$. More specifically, each element can be written as:

(25) $$\begin{array}{ccc} M_{ij}= & [p_{00-i}^{t}q_{00-j}^{t},\cdots ,p_{0\eta -i}^{t}q_{0\eta -j}^{t},p_{11-i}^{t}q_{11-j}^{t},\cdots , & p_{1\eta -i}^{t}q_{1\eta -j}^{t},\cdots ,p_{\eta 0-i}^{t}q_{\eta 0-j}^{t},\cdots ,p_{\eta \eta -i}^{t}q_{\eta \eta -j}^{t}{]}^{T}. \end{array}$$

Suppose that the stable vector of $M_{d}$ is $v_{d}$, we have $v_{d}^{T}M_{d}=v_{d}^{T}$, and the expected utilities of the requestor and the worker are $E_{r}=v_{d}^{T}W_{r}$ and $E_{w}=v_{d}^{T}W_{w}$, respectively.

Assuming that $M_{d}^{\prime }=M_{d}-I$, we have $v_{d}^{T}M_{d}^{\prime }=0$. With the same calculation as that under the discrete model, we obtain that $v_{d}^{T}$ is proportional to each row of $Adj(M_{d}^{\prime })$. Therefore, for any vector $f=[f_{00},f_{01},\cdots ,f_{\eta \eta }]$, with the known condition $\sum_{j=0}^{\eta }q_{i-j}^{t}=1$, one can compute its dot product with $v_{d}$ as follows:

(26) $$\begin{array}{cc} v_{d}\cdot f & =D(p^{t},q^{t},f),\\ & =det\left[\begin{array}{cccc} p_{00-0}^{t}q_{00-0}^{t} & \cdots & p_{00-\eta }^{t} & f_{00}\\ \vdots & \vdots & \vdots & \vdots \\ p_{(\eta -1)\eta -0}^{t}q_{(\eta -1)\eta -0}^{t} & \cdots & p_{(\eta -1)\eta -\eta }^{t} & f_{(\eta -1)\eta }\\ p_{\eta \eta -0}^{t}q_{\eta \eta -0}^{t} & \cdots & p_{\eta \eta -\eta }^{t}-1 & f_{\eta 0}\\ \vdots & \vdots & \vdots & \vdots \\ p_{\eta \eta -0}^{t}q_{\eta \eta -0}^{t} & \cdots & p_{\eta \eta -\eta }^{t}-1 & f_{\eta \eta } \end{array}\right]. \end{array}$$

It is clear that the penultimate column of (26) can be decided only by the requestor, denoted as ${\tilde{p}}_{\left(\eta +1)(\eta +1\right)}$. When $f=\alpha W_{r}+\beta W_{w}+\gamma 1$, we have $v_{d}^{T}\cdot f=v_{d}^{T}\left(\alpha W_{r}+\beta W_{w}+\gamma \right)=\alpha E_{r}^{t}+\beta E_{w}^{t}+\gamma$. Therefore, if ${\tilde{p}}_{\left(\eta +1)(\eta +1\right)}=\chi \left(\alpha W_{r}+\beta W_{w}+\gamma 1\right)$, we have $\alpha E_{r}^{t}+\beta E_{w}^{t}+\gamma =0$. When the small number δ approaches to 0, the above lemma is proved. ☐

The weighted *social welfare* is defined as $E_{all}=\alpha E_{r}+\beta E_{w}=-\gamma$. Then according to Lemma 1, the requestor’s strategy $p^{t}\left(x_{-1},y_{-1},x\right)$ is the only factor affecting the weighted social welfare $E_{all}$, which can be regarded as the zero-determinant strategy in the continuous model. To be specific, the requestor can solve the following optimization problem to achieve a unilateral control on the social welfare without considering the worker’s strategy:

(27) $$\begin{array}{cc} \max & \;\;E_{all}=\alpha E_{r}\left(p^{t},q^{t}\right)+\beta E_{w}\left(p^{t},q^{t}\right),\forall q^{t},\\ s.t. & \left\{\begin{array}{c} 0\leq p^{t}\left(x_{-1},y_{-1},x\right)\leq 1,\\ \alpha E_{r}+\beta E_{w}+\gamma =0. \end{array}\right. \end{array}$$

Let $W\left(x,y\right)=\alpha w_{r}\left(x,y\right)+\beta w_{w}\left(x,y\right)$; then the above problem can be converted into

(28) $$\begin{array}{cc} \min & \;\;\gamma ,\\ s.t. & \left\{\begin{array}{c} 0\leq p^{t}\left(x_{-1},y_{-1},x\right)\leq 1,\\ p^{t}\left(x_{-1},y_{-1},x\right)=\chi \left(W\left(x,y\right)+\gamma \right),\\ \chi \neq 0. \end{array}\right. \end{array}$$

For the case of χ>0, considering the constraint $p^{t}\geq 0$, we have

(29) $$\begin{array}{cc} \gamma_{min} & =\max\left(\Gamma \left(x^{\prime },y^{\prime }\right)\right),\forall x^{\prime }\in \left[l_{r},h_{r}\right],\forall y^{\prime }\in \left[l_{w},h_{w}\right],\\ \Gamma (x^{\prime },y^{\prime }) & =\left\{\begin{array}{cc} -W(x^{\prime },y^{\prime }), & x^{\prime }<h_{r},\\ -W\left(x^{\prime },y^{\prime }\right)-\frac{1}{\chi }, & x^{\prime }=h_{r}. \end{array}\right. \end{array}$$

While considering the constraint condition $p^{t}\leq 1$, we have

(30) $$\begin{array}{cc} \gamma_{max} & =\min\left(\Gamma \left(x^{\prime \prime },y^{\prime \prime }\right)\right),\forall x^{\prime \prime }\in \left[l_{r},h_{r}\right],\forall y^{\prime \prime }\in \left[l_{w},h_{w}\right],\\ \Gamma (x^{\prime \prime },y^{\prime \prime }) & =\left\{\begin{array}{cc} -W\left(x^{\prime \prime },y^{\prime \prime }\right)+\frac{1}{\chi }, & x^{\prime \prime }<h_{r},\\ -W(x^{\prime \prime },y^{\prime \prime }), & x^{\prime \prime }=h_{r}. \end{array}\right. \end{array}$$

When $\gamma_{min}<\gamma_{max}$, γ has a feasible solution. In other words, $\max\left(\Gamma \left(x^{\prime },y^{\prime }\right)\right)<\min\left(\Gamma \left(x^{\prime \prime },y^{\prime \prime }\right)\right),\forall x^{\prime },x^{\prime \prime }\in \left[l_{r},h_{r}\right],\forall y^{\prime },y^{\prime \prime }\in \left[l_{w},h_{w}\right]$. Since χ could be any positive number, we can get the minimum value of γ:

(31) $$\gamma_{min}=\max\left(-W\left(x^{\prime },y^{\prime }\right)\right),\forall x^{\prime }\in \left[l_{r},h_{r}\right),\forall y^{\prime }\in \left[l_{w},h_{w}\right].$$

For the case of χ<0, when considering $p^{t}\geq 0$, we have $\gamma_{min}=\max\left(\Gamma \left(x^{\prime \prime },y^{\prime \prime }\right)\right),\forall x^{\prime \prime }\in \left[l_{r},h_{r}\right],\forall y^{\prime \prime }\in \left[l_{w},h_{w}\right]$; while when considering $p^{t}\leq 1$, we have $\gamma_{max}=\min\left(\Gamma \left(x^{\prime },y^{\prime }\right)\right),\forall x^{\prime }\in \left[l_{r},h_{r}\right],\forall y^{\prime }\in \left[l_{w},h_{w}\right]$. Then, γ is feasible only when $\gamma_{min}<\gamma_{max}$, i.e., $\max\left(\Gamma \left(x^{\prime \prime },y^{\prime \prime }\right)\right)<\min\left(\Gamma \left(x^{\prime },y^{\prime }\right)\right),\forall x^{\prime },x^{\prime \prime }\in \left[l_{r},h_{r}\right],\forall y^{\prime },y^{\prime \prime }\in \left[l_{w},h_{w}\right]$. Thus, we can obtain the following result:

(32) $$\gamma_{min}=\max\left(-W\left(h_{r},y^{\prime \prime }\right)\right),\forall y^{\prime \prime }\in \left[l_{w},h_{w}\right].$$

Then, according to (31) and (32), the requestor’s zero-determinant strategy $p^{t}$ can be calculated as follows:

(33) $$\begin{array}{c} p^{t}\left(x_{-1},y_{-1},h_{r}\right)=\left\{\begin{array}{cc} \chi (W\left(x_{-1},y_{-1}\right)+\gamma_{min}), & x_{-1}<h_{r},\\ \chi (W\left(x_{-1},y_{-1}\right)+\gamma_{min})+1, & x_{-1}=h_{r}. \end{array}\right. \end{array}$$

## 4. Evaluation of the Proposed Schemes

In this section, we evaluate the zero-determinant strategy based schemes proposed in Section 3 by simulations in Matlab. Considering the general definition of social welfare, we set α=β=1. First, we investigate the scheme proposed for the discrete model and report the results for the parameter settings $R_{w}=3,R_{r}=3,a=3,b=2,m=3,n=2$, which implies that $S_{r}=\left(3,0,5,2\right)$ and $S_{w}=\left(3,5,0,2\right)$. Note that we also simulate other parameter settings satisfying the relationship among these parameters mentioned in Section 2, and obtain very similar results, which are omitted due to page limit.

**Figure 2:**
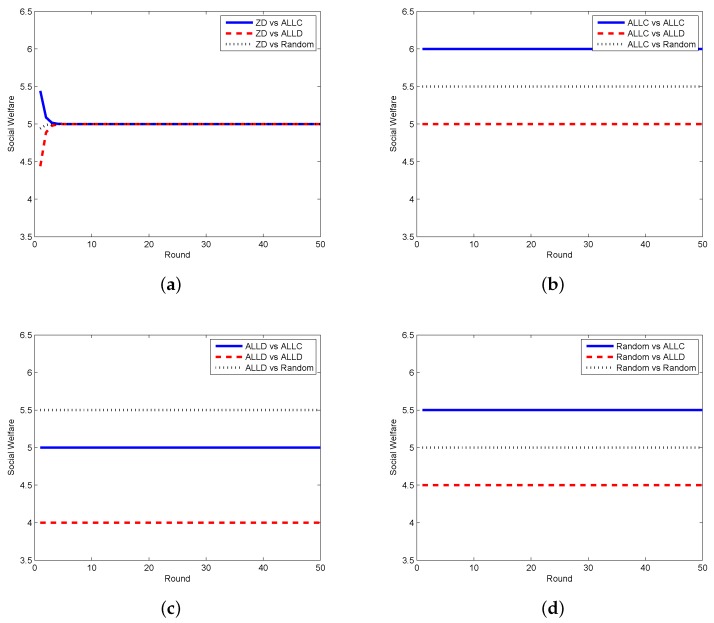
Social welfare with different strategy pairs (requestor vs. worker) among ZD, ALLC, ALLD, and Random in the discrete model. (**a**) requestor adopts the ZD strategy; (**b**) requestor adopts the ALLC strategy; (**c**) requestor adopts the ALLD strategy; (**d**) requestor adopts the Random strategy.

**Figure 3:**
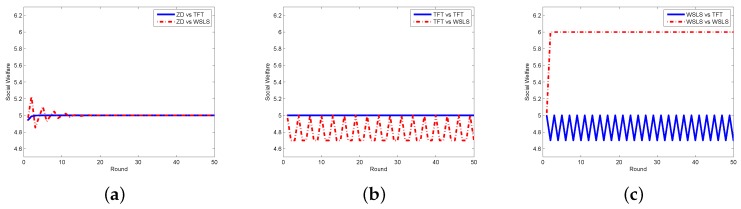
Social welfare with different strategy pairs (requestor vs. worker) among ZD, TFT, and WSLS in the discrete model. (**a**) requestor adopts the ZD strategy; (**b**) requestor adopts the TFT strategy; and (**c**) requestor adopts the WSLS strategy.

In order to testify the effectiveness of our proposed scheme, we compare the zero-determinant strategy with five other classical strategies that might be adopted by the requestor. In Figure 2, we display the results when the requestor adopts the proposed zero-determinant (ZD), all cooperation ($p^{t}=\left[1,1,1,1\right]$, denoted as ALLC), all defection ($p^{t}=\left[0,0,0,0\right]$, denoted as ALLD), and random ($p^{t}=\left[0.5,0.5,0.5,0.5\right]$, denoted as Random) strategies while the worker adopts three strategies, i.e., ALLC, ALLD, and Random. It can be seen that the social welfare can be kept stable and can achieve its maximum value when the requestor adopts the ZD strategy and the worker adopts any strategy. However, when the requestor takes the other three strategies, the social welfare is determined by the strategies of both the requestor and the worker, which means that the requestor has no dominance on the control over the social welfare. The comparison results between the ZD strategy and two other classical strategies, i.e., tit-for-tat (TFT) and win-stay-lose-shift (WSLS), are presented in Figure 3. It is clear that when the requestor adopts the ZD strategy and the worker takes either TFT or WSLS, the social welfare is approximately the same and is stable; while when the requestor changes to any other strategy, the social welfare is fluctuated most of the time and is affected by the strategies of both parties.

**Figure 4:**
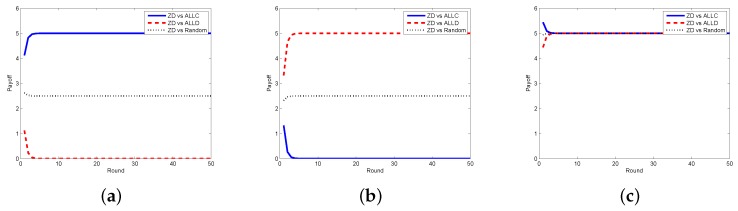
Payoffs with ZD vs. ALLC/ALLD/Random in the discrete model. (**a**) requestor’s payoff; (**b**) worker’s payoff; (**c**) social welfare.

**Figure 5:**
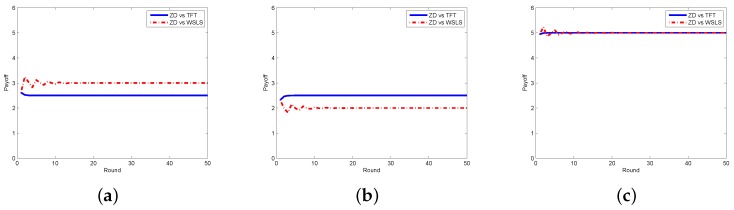
Payoffs with ZD vs. TFT/WSLS in the discrete model. (**a**) requestor’s payoff; (**b**) worker’s payoff; (**c**) social welfare.

Next, we look at the respective payoffs of the requestor and the worker, depicted together with the total payoff (i.e., social welfare). The results when the requestor adopts the ZD strategy and the worker takes ALLC, ALLD, and Random strategies are shown in Figure 4, from which one can find out that the payoffs of the requestor and the worker are getting stable as the number of rounds increases, so does the social welfare. In Figure 5, we plot the payoffs of the requestor and the worker as well as the social welfare at each round under different strategy pairs, i.e., ZD versus TFT and ZD versus WSLS. The results indicate that the payoffs gradually become stable, which is consistent with the change of the social welfare. From the above two figures, one can also find out that the requestor’s payoff in stable state is no less than that of the worker in all strategy pairs except for the case when the requestor takes the ZD strategy while the worker takes the ALLD strategy, which seems to be unwise for a reasonable worker in practice.

**Figure 6:**
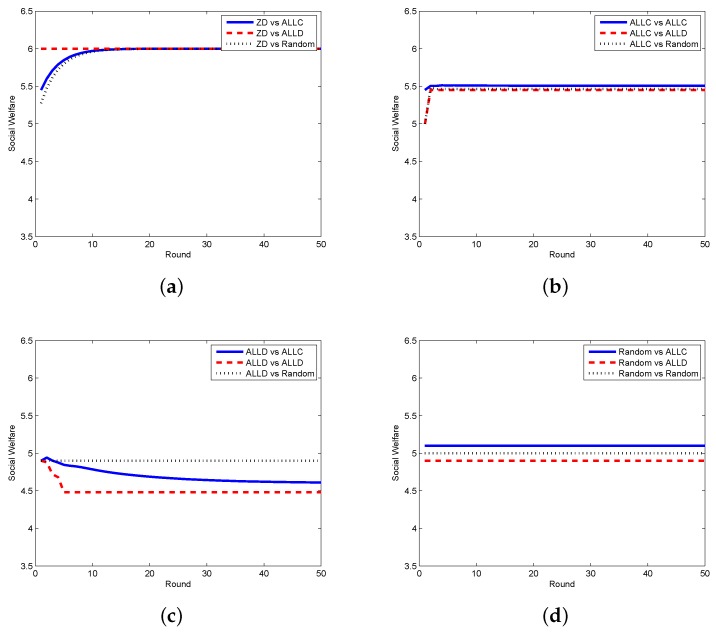
Social welfare with different strategy pairs (requestor vs. worker) among ZD, ALLC, ALLD, and Random in the continuous model. (**a**) requestor adopts the ZD strategy; (**b**) requestor adopts the ALLC strategy; (**c**) requestor adopts the ALLD strategy; (**d**) requestor adopts the Random strategy.

**Figure 7:**
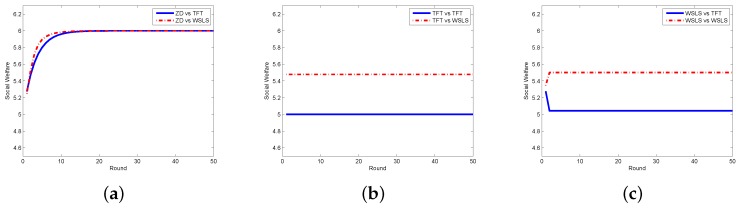
Social welfare with different strategy pairs (requestor vs. worker) among ZD, TFT, and WSLS in the continuous model. (**a**) requestor adopts the ZD strategy; (**b**) requestor adopts the TFT strategy; and the (**c**) requestor adopts the WSLS strategy.

In the scenario of continuous strategies adopted by both the requestor and the worker, we assume that they can choose their strategies from [0,10], i.e., $l_{r}=l_{w}=0,h_{r}=h_{r}=10$. Suppose that $\phi \left(y\right)=\frac{1}{1+exp(-y)},A_{r}=4,B_{r}=0.1$, and $\psi \left(y\right)=\frac{1}{1+exp(-y)}-1,A_{w}=0.3,B_{w}=4$; thus we have $w_{r}\left(0,0\right)=w_{w}\left(0,0\right)=2$ and $w_{r}\left(10,10\right)=w_{w}\left(10,10\right)=3$. We first compare the ZD strategy with the ALLC strategy corresponding to $p^{t}\left(x_{-1},y_{-1},h_{r}\right)=1$, the ALLD strategy corresponding to $p^{t}\left(x_{-1},y_{-1},h_{r}\right)=0$, and the Random strategy corresponding to $p^{t}\left(x_{-1},y_{-1},x\right)=1/\left(h_{r}-l_{r}\right)$, applied by the requestor. As shown in Figure 6, when the requestor adopts the ZD strategy, the social welfare becomes stable no matter whether the worker adopts the ALLC strategy ($q^{t}\left(x_{-1},y_{-1},h_{w}\right)=1$), the ALLD strategy ($q^{t}\left(x_{-1},y_{-1},h_{w}\right)=0$), or the Random strategy ($q^{t}\left(x_{-1},y_{-1},y\right)=1/\left(h_{w}-l_{w}\right)$). However, when the requestor employs any other classical strategy, the social welfare is related to the strategies of both sides. When TFT and WSLS strategies are employed, the results are presented in Figure 7. It can be seen that when the requestor takes the ZD strategy, no matter what strategies (TFT or WSLS) the worker adopts, the social welfare stays in the same stable value. However, when the requestor adopts either TFT or WSLS , the social welfare is also affected by the worker’s strategy. Furthermore, from the above two figures, one can see that even though the social welfare with some other pairs of strategies can reach stability, it is always less than that can be achieved when the requestor adopts the ZD strategy.

**Figure 8:**
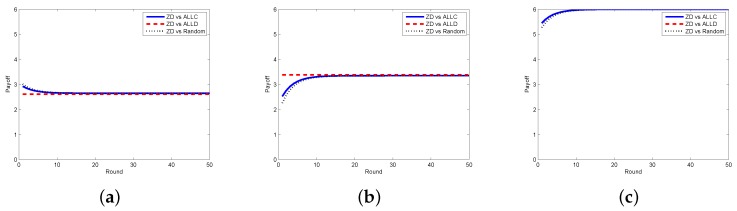
Payoffs with ZD vs. ALLC/ALLD/Random in the continuous model. (**a**) requestor’s payoff; (**b**) worker’s payoff; (**c**) social welfare.

**Figure 9:**
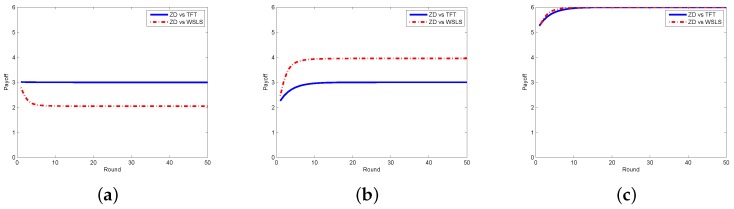
Payoffs with ZD vs. TFT/WSLS in the continuous model. (**a**) requestor’s payoff; (**b**) worker’s payoff; (**c**) social welfare.

We also examine the payoffs of the requestor and the worker, as well as the total payoff (i.e., social welfare), under different strategy pairs they adopt. When the requestor adopts the ZD strategy and the worker adopts ALLC, ALLD, or Random, the results are reported in Figure 8, which clearly shows that the payoffs of the requestor and the worker are getting stable when the number of rounds increases, so does the social welfare. Figure 9 reports the payoffs of the requestor and the worker as well as the social welfare in each round under different strategy pairs (i.e., ZD versus TFT and ZD versus WSLS). One can see that the payoffs and the social welfare are gradually becoming stable.

## 5. Related Work

Recently, a large number of works focusing on devising incentive schemes to motivate workers’ participation in mobile crowdsensing have been proposed. Generally speaking, there are three typical classes of incentives, i.e., *entertainment, service, and money*. Entertainment-based incentive schemes are always realized by delicately designing games with attractive rewarding and penalizing strategies. In [12], Jordan et al. developed a location-based game called *Ostereiersuche* to collect the landmark structural information by encouraging users to search for coupons with some navigation hints, which is certainly an entertainment based incentive mechanism. Hoh et al. [13] proposed *TruCentive*, a service incentive mechanism that employs the parking information as an incentive to stimulate the drivers to report available parking time and places, by which the reporters could obtain corresponding points for future parking information requests. Lan et al. [14,15] designed a virtual credit based incentive mechanism for collecting mobile surveillance data, by which a participant could download data only when he/she paid the amount of required virtual credit earned by uploading sensing data or sharing bandwidth with others. The monetary incentives mostly rely on an economics tool, i.e., auction. In [16], considering the opportunistic occurrences in the places of interest, several online reverse-auction-based incentive mechanisms were put forward to achieve desired computation efficiency, individual rationality, profitability, and truthfulness in mobile crowdsensing. Chen et al. in [17] also took account of the participants’ dynamic arriving, based on which they devised a truthful online auction mechanism with no requirement on the previous knowledge.

Considering that data quality is an essential factor affecting the performance of mobile crowdsensing, more and more literature have turned to tackling the challenges of low task quality offered by participants. In [7], a metric called *quality of information (QoI)* was considered in the design of the incentive mechanisms for mobile crowdsensing and a reverse combinatorial auction was adopted to realize a maximized social welfare for the requestor and all the participating workers. Each mobile crowdsensing task was distributed by the requestor according to the bids of the workers along with the sum of QoI they can provide, which was obviously an NP-hard (non-deterministic polynomial-time hard) problem and could be only approximately solved. Another auction based and data quality accounted research work was reported in [8], in which an algorithm based on reverse Vickrey auction was proposed to maximize the social welfare when choosing workers in mobile crowdsensing, where the concept of data quality was mainly determined by the data type provided by a specific worker. After the subset of winning workers was selected by the QDA algorithm, the independent payment for each winner could be derived, along with some extra rewards proportional to the number of requestors adopting the submitted sensing data. In order to further protect the crowdsensing system from fake data attack during the auction-based working process, Xiao et al. [18] utilized game theory to accommodate the data quality into the utility functions of the requestor and the workers, and then derived the Nash Equilibrium according to the best responses. To be specific, all the submitted sensing data were classified into several types indicating different data quality levels as well as different payment levels, where the fake-data providers would be rated to be the lowest level and given the lowest payment of zero.

Note that even though auction based schemes are effective for data quality sensitive situations, such an approach could be unnecessary and burdensome for some simple mobile crowdsensing applications with low limitations on budget or with strict requirement on promptness.

Different from the above schemes that evaluate the data quality after completing the receiving process, data quality can be estimated in advance to help determine the best subset of workers undertaking the sensing tasks. For example, an Expectation Maximization algorithm and Bayesian inference were jointly taken by [19] to achieve data quality assurance in mobile crowdsensing: with the estimated data quality, a relatively fair and appropriate payoff for each worker can be calculated through a contribution quantification process with a *Information Theory* based model; and at the end of each task round, the estimation of the data quality provided by each worker is updated so as to evaluate data quality more precisely in the next round.

With the historical execution behavior of workers, some reputation based methods were proposed to settle the low quality problem of submitted data in mobile crowdsensing. In [9], Ref. Amintoosi et al. put forward a reputation framework for social participatory crowdsensing systems, rating workers via a fuzzy inference system according to their contribution quality and social trustworthiness level. After earning this reputation score, they utilized the PageRank algorithm to filter out the low quality data contributors online.

In addition, historical records of the system feedback can also offer a possibility for some data analysis techniques to improve the data quality in mobile crowdsensing. Kawajiri et al. [10] proposed a framework named *Steered Crowdsensing* based on some commercial location-based services (LBSs) to directly increase the job quality provided by the workers in crowdsensing, especially in opportunistic crowdsensing scenario. A *quality indicator* was introduced into the machine learning settings to optimally determine the payment of each sensing task, corresponding to a specific location, in a feedback-system manner, and further determine the quality of services the whole crowdsensing system can offer. This implies that there is a clear display of payment for each subtask at different locations, and the workers only need to make decisions on whether or not to complete the data sensing task in the required places.

On the other hand, the embedded sensors in the workers’s devices can not only help collect data in mobile crowdsensing but also potentially leak private information of the workers, such as location privacy indicated by GPS. Thus, the tradeoff between privacy preservation and data contribution of the workers plays an important role in the stable and prosperous development of mobile crowdsensing, which has been widely investigated in recent years [20,21,22]. In [20], Alsheikh et al. proposed a mechanism based on Vickrey–Clarke–Groves auction, which can guarantee data accuracy of mobile crowdsensing applications and simultaneously protect the workers’ privacy according to their requirements. Furthermore, in [22], Gisdakis et al. extended their basic security and privacy framework called SPPEAR [21] to resist as many attacks as possible, which could support various incentive mechanisms for participants in mobile crowdsensing.

From the above discussions, one can see that existing research related to mobile crowdsensing mainly focuses on incentive mechanism design and data quality control, as well as privacy and security protection. Incentive mechanisms are mostly based on providing entertainment, services, or money, where money based incentives always rely on auctions, while data quality control schemes mainly take advantage of auctions, game theory, data mining, and data analysis methods (e.g., Expectation Maximization algorithm, machine learning). The privacy and security issues are attracting more and more attention in recent years, which can be tackled by truthful actions and/or delicately designed security systems.

## 6. Conclusions

In this paper, we investigate the issue of social welfare control in crowdsensing. With the help of zero-determinant strategy, we propose a powerful approach for the requestor to unilaterally control the social welfare, i.e., the sum of the expected payoffs of the requestor and the worker, with no consideration on the strategy of the worker under both the discrete-strategy model and the continuous-strategy model. More specifically, with a delicate computation of her strategy, the requestor can achieve maximized and stable social welfare regardless of the worker’s strategy and complete the payment process at the same time. The simulation results validate the effectiveness of our proposed mechanisms, showing that the requestor adopting zero-determinant strategy can ensure the social welfare to stay at a desired level on her own.
